# microRNA Let‐7b: A Novel treatment for endometriosis

**DOI:** 10.1111/jcmm.13807

**Published:** 2018-07-31

**Authors:** Cagdas Sahin, Ramanaiah Mamillapalli, Kyong W. Yi, Hugh S. Taylor

**Affiliations:** ^1^ Department of Obstetrics, Gynecology and Reproductive Sciences Yale School of Medicine New Haven Connecticut

**Keywords:** endometriosis, microRNA, miRNA Let‐7b, oligonucleotide treatment

## Abstract

Endometriosis is an oestrogen‐dependent, chronic inflammatory disease that affects 10% of reproductive‐aged women. Current treatment options depend on female sex steroid hormone modulation; however, all have side effects and are not useful in women who want to conceive. microRNAs treatments have provided promising results for some chronic diseases and cancers. We have previously shown the microRNA Let‐7b is repressed in endometriosis and that loss of Let‐7 contributes to the pathophysiology of the disease. Here, we propose using microRNA Let‐7b for the treatment of endometriosis in a murine model. Endometriosis was treated using microRNA Let‐7b or a scrambled control microRNA. Let‐7b treatment resulted in reduced endometriosis lesion size. Decreased gene expression was noted in several genes known to promote endometriosis growth including ER‐α, ER‐ß, Cyp19a, KRAS 4A, KRAS 4B and IL‐6. These results indicate that microRNA Let‐7b has a pleiotropic role in endometriosis pathophysiology affecting oestrogen signalling, inflammation and growth factor receptors. Local treatment of endometriosis with Let‐7b is a promising therapy for endometriosis that simultaneously affects multiple pathways driving endometriosis without systemic hormonal side effects.

## 1 INTRODUCTION

Endometriosis has many symptoms that negatively affect both the reproductive capability and professional/social life of affected women.^1^ This disease is characterized by the proliferation and growth of endometrial tissue outside of the uterine cavity that causes pelvic pain and infertility.^2^, ^3^ The incidence of the endometriosis is approximately 10% in reproductive‐aged women and 20%‐50% of women with infertility or pelvic pain.^4^ The pathophysiology of endometriosis is not well understood; therefore, current treatment options for endometriosis are limited to manipulation of female sex steroid hormones.^5^, ^6^ These treatment options have significant side effects and are all contraindicated in women wishing to conceive. Nonhormonal therapies that specifically target the endometriosis are needed.

microRNAs (miRNAs) are small noncoding functional RNA molecules approximately 22 nucleotides in length. They play a critical role in the regulation of gene expression by binding directly to the 3′‐untranslated region (3′‐UTR) of messenger RNA (mRNA) in a sequence‐specific fashion, blocking translation or leading to mRNA degradation.^7^ miRNAs have a major role in regulation of development and in cellular homeostasis.^8^, ^9^ Additionally, aberrant microRNA expression is linked to many diseases such as cancer,^10^ cardiovascular disorders^11^ and inflammatory diseases.^12^ microRNAs are the potent regulators of gene expression in endometriosis.^13^ Distinct miRNA expression profiles have been identified in microarray studies of eutopic and ectopic endometrial tissue samples.^13^ We previously reported that circulating microRNAs, including Let‐7b‐5p, were significantly decreased in the serum of patients with endometriosis as well as animal models of the disease.^14^, ^15^ Further, we have reported decreased Let‐7 family members in endometriosis tissue where they are involved in the regulation of genes such as KRAS and aromatase.^16^, ^17^ These studies prompted us to hypothesize that miRNA Let‐7b‐5p might have a major role in the pathogenesis and treatment of endometriosis. Here, we report the therapeutic use of miRNA Let‐7b‐5p in the treatment of endometriosis in a murine model.

## 2 MATERIALS AND METHODS

### 2.1 Animals

Six‐ to eight‐week‐old C57BL/6J wild‐type female mice were purchased from Jackson Laboratories (Bar Harbor, ME, USA). Mice were maintained in the animal facility of Yale School of Medicine. They housed five animals per cage in a 12‐hour light, 12‐hour dark cycle (7 am‐7 pm) with ad libitum access to food and water. All animals were treated under an approved protocol by Yale University Institutional Animal Care and Use Committee. Mice were acclimated at least 1 week, and vaginal cytology analysis was performed to determine oestrous cycle stage of individual animals prior to surgery. Ten animals that were in diestrous stage were used as recipient, and three mice that were in oestrus stage were used as donors.

### 2.2 Induction of endometriosis in mice

Endometriosis was induced in ten mice using a modified version of the syngeneic endometriosis protocol that has been used previously in our laboratory.^18^ In accordance with this model, identical sizes of uterine tissue fragments were sutured onto the peritoneal surface. Three mice in oestrus stage were killed using a CO_2_ chamber, both uterine horns from each mouse were removed and opened longitudinally and divided into equal fragments measuring 4 mm^2^. These fragments were preserved on ice in DMEM/F12 Ham 1:1 media (Gibco; Grand Island, NY, USA) until transplantation. For implantation, ten mice were anaesthetized by inhalation of isoflurane (Isothesia; Henry Schein, OH, USA) and laparotomy was performed by midline incision. Two uterine fragments were sutured on each right and left peritoneal surface area using 5‐0 polyglactin sutures (Vicryl; Ethicon, Somerville, NJ, USA). Subsequently, the peritoneum and skin were closed with same sutures.

### 2.3 microRNA Let‐7b‐5p treatment

Ten animals with experimentally induced endometriosis were randomly divided into two groups of five mice in each. Two weeks after the induction of endometriosis, miRNA Let‐7b‐5p treatment was initiated with Let‐7b‐5p mimic (UGAGGUAGUAGGUUGUGUGGUU‐ mirBase accession number: MIMAT0000522) and miRNA cel‐miR‐67‐3p miRNA (UCACAACCUCCUAGAAAGAGUAGA‐ mirBase accession number: MIMAT0000039) as a control. These miRNAs were purchased from W. M. Keck Oligonucleotide Synthesis Facility (Yale University, New Haven, CT, USA). miRNAs were injected into the peritoneal cavity by in vivo‐jetPEI carrier (Polyplus‐transfection, Illkirch, France). The oligonucleotide + in vivo‐jetPEI mixture was prepared according to the manufacturer's guidelines for intraperitoneal oligonucleotide injection. Accordingly, 1.0 mL 5% glucose mixture including 100 μg nucleic acid and 16 μL carrier reagent (N/P = 8) was prepared for each injection, and mice were treated by intraperitoneal injections for every 3 days for 2 weeks. The dose chosen was based on our previously described in vitro dose–response experiments.^17^

### 2.4 Macroscopic and microscopic evaluation of lesions

After 2 weeks of treatment with Let‐7b‐5p miRNA, animals were killed within a CO2 chamber and endometriotic lesions were removed from peritoneum of the mice. All lesions were individually measured, and lesion's volumes were calculated with using smallest diameter^2^ × largest diameter/2 formula. Half of each lesion was kept in RNA stabilization solution (RNA later; Qiagen, Hilden, Germany) for mRNA isolation to determine the gene expression by qRT‐PCR analysis, and the other half was kept in 4% paraformaldehyde solution for immunohistochemistry studies. After H&E staining, all lesions were evaluated under light microscope to confirm endometriosis. The area of each lesion was calculated using the NIS Elements Imaging software program (3.10, Nikon, Brighton, MI, USA).

### 2.5 RNA extraction and quantitative real‐time polymerase chain reaction (qRT‐PCR)

Endometriotic lesions were thawed on ice and minced into fine pieces and homogenized in 1.0 mL of TRIzol reagent (Invitrogen, Carlsbad, CA, USA), RNA chloroform extracted and precipitated in isopropyl alcohol and dissolved in 30 μL of RNase‐free water. The total RNA was purified using the RNeasy cleanup kit (Qiagen, Valencia, CA, USA), according to the manufacturer's protocol, treated using recombinant shrimp DNase (USB, Cleveland, OH, USA) to eliminate DNA contamination and quantified by a NanoDrop spectrophotometer. Purified RNA was immediately used for cDNA synthesis or stored at −80°C until use later. For cDNA synthesis, purified RNA (1000 ng) was reverse‐transcribed using iScript cDNA synthesis kit (Bio‐Rad Laboratories, Hercules, CA, USA). Real‐time quantitative PCR (real‐time qPCR) was performed using SYBR Green (Bio‐Rad) and optimized in the MyiQ single‐color real‐time PCR detection system (Bio‐Rad). Primer sequences used for gene expression are listed in Table 1. The specificity of the amplified transcript and absence of primer dimers were confirmed by a melting curve analysis. Gene expression was normalized to that of β‐actin as an internal control. Relative mRNA expression was calculated using the comparative cycle threshold (Ct) method (2^−ΔCT^).^19^, ^20^ All experiments were carried out three times and each in duplicate.

**Table 1:** Primer sequences used for gene expression by qRT‐PCR

Gene	Forward sequence	Reverse sequence
*ER‐*α	TCTGCCAAGGAGACTCGCTACTGT	GCTTGGCCAAAGGTTGGCAG
*ER‐ß*	GCCAACCTCCTGATGCTTCTTT	TTGTACCCTCGAAGCGTGTGA
*CYP19A1*	CTTGGCTGTAGGGGGCATAC	GCGCTATTTGGCCTGAGTTG
*KRAS4A*	AGATGTGCCTATGGTCCTGGTAG	CAATCTGTACTGTCGGATCTCTCTC
*KRAS4B*	GATGTGCCTATGGTCCTGGTAG	CATCGTCAACACCCTGTCTTG
*IL‐6*	TAGTCCTTCCTACCCCAATTTCC	TTGGTCCTTAGCCACTCCTTC
*IGF‐1*	GGTGGTTTATGAATGGTT	AGGGTGTGTCTAATGGAG
*Cyclin‐D1*	AAGTGCGTGCAGAAGGAGATTGT	GGATAGAGTTGTCAGTGTAGATGC
*MMP‐2*	CCCTCAAGAAGATGCAGAAGTTC	TCTTGGCTTCCGCATGGT
*TLR‐4*	TTCAGAACTTCAGTGGCTGGATT	CCATGCCTTGTCTTCAATTGTTT
*IL‐10*	GCTGCGGACTGCCTTCAG	AGGAGTCGGTTAGCAGTATGTTGTC
*ß‐actin*	AGTGTGACGTTGACATCCGTA	GCCAGAGCAGTAATCTCCTTCT

### 2.6 Immunohistochemistry analysis

Lesions were fixed in 4% paraformaldehyde and embedded in paraffin. Five‐micrometre tissue sections were mounted on slides followed by 15 minutes of boiling in sodium citrate (pH 6) for antigen retrieval and blocked with 10% goat serum (Vector Laboratories, Burlingame, CA, USA). Slides were incubated at 4°C overnight with anti‐ER‐α (1:300; sc‐542; Santa Cruz Biotechnology, Inc., Dallas, TX, USA), anti‐ER‐ß (1:500; sc‐8974; Santa Cruz Biotechnology, Inc.), anti‐aromatase (1:700; ab‐18995; Abcam Inc., USA) and anti‐KRAS (1:400; ab‐216890; Abcam Inc., Cambridge, MA, USA) primary antibodies to determine protein expression, respectively. Slides were incubated 60 minutes at room temperature with biotinylated goat anti‐rabbit IgG (1:200; Vector Laboratories), and for detection, ABC Vectorstain Elite reagents with DAB plus H2O2 (Vector Laboratories) were used. Tissue sections were counterstained with haematoxylin (Sigma‐Aldrich, St. Louis, MO, USA). Images of stained sections were captured using Nikon Eclipse 80i microscope (Nikon).

### 2.7 Statistical analysis

GraphPad Prism 7.0 a software (GraphPad Software, La Jolla, CA, USA) was used for all statistical analyses. All in vitro experiments were performed in triplicate, and the mean for each individual animal was used for statistical analysis. The quantitative data were tested for normality using the Shapiro‐Wilk test. Non‐normally distributed continuous variables were compared using Mann‐Whitney *U* test. Student *t* test was used for evaluating of normally distributed variables. *P* < 0.05 was considered as statistically significant.

## 3 RESULTS

### 3.1 Let‐7b treatment and evaluation of lesions

**Figure 1:**
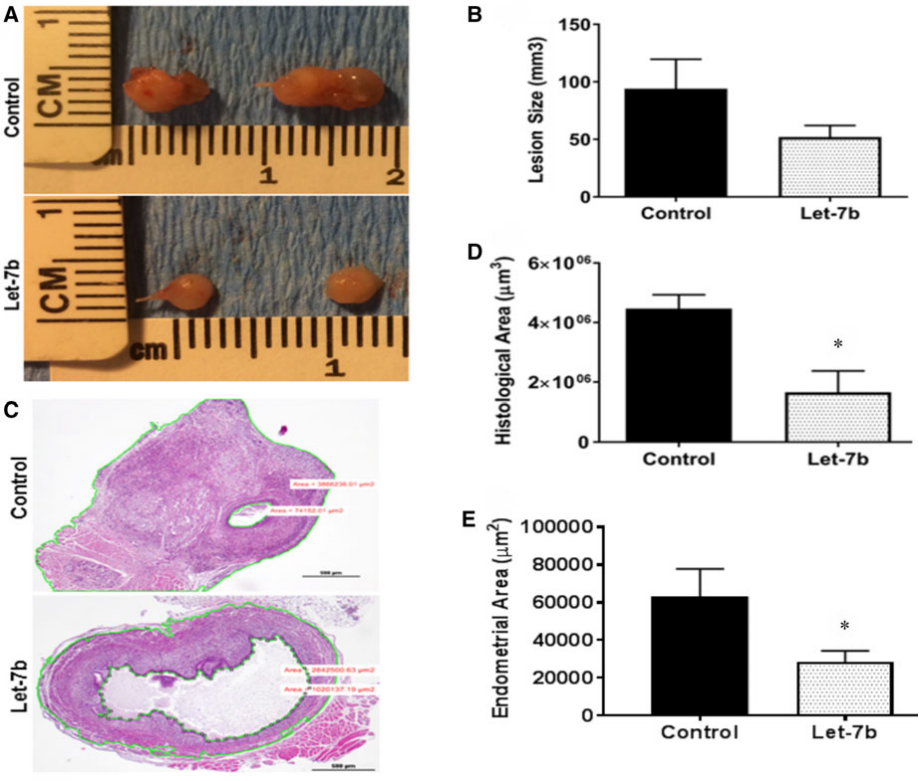
Macroscopic and microscopic evaluation of lesions. A, Macroscopic images of lesions from Let‐7b‐treated and untreated groups. B, Comparison of total lesion size between the two groups, including fluid‐filled cystic areas. C, The histologic area of actual endometriosis excludes the fluid‐filled cystic area. The area between the outer and inner green lines was used to calculate the histologic area of lesions. D, Comparison of histologic area of lesions; n = 5 mice per group. E, Difference in mouse endometrial tissue area between Let‐7b‐treated and control mice (n = 5). Data are presented as mean percentage ± SEM.; *P = 0.03

No adverse reactions of the Let‐7 treatment were noted. At the end of the miRNA, Let‐7b‐5p treatment period (2 weeks) mice were killed and endometriotic lesions collected. We first compared lesion size and volume between the Let‐7 treated and control groups. All of the lesions were cystic and the fluid‐filled portion responded little to this short‐term treatment. Gross lesion size was lower in the Let‐7b treated group; however, the difference was not significant (*P* = 0.14) (Figure 1A & B). Additionally, lesions were compared by measuring histological area of actual endometriosis. The histologic area was evaluated under the microscope to exclude the cystic part of lesions; the fluid‐filled cystic area was subtracted from the total lesion are to determine the amount of active endometriosis. A significantly diminished histologic endometriosis tissue area was observed in the Let‐7b treatment group (*P* = 0.03) (Figure 1C & D) compared to the control group. The histological area of endometriosis lesions included the grafted myometrium in Let‐7b‐treated and control mice. We also specifically measured the area of endometrial tissue and determined that it was also significantly reduced in Let‐7b‐treated mice compared to control mice, as shown in Figure 1E.

### 3.2 Differential expression of genes that are involved in endometriosis

**Figure 2:**
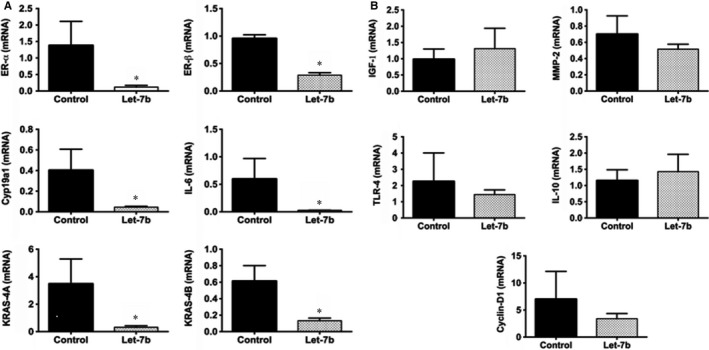
Effect of Let‐7b treatment on mRNA expression of selected genes involved in the pathophysiology of endometriosis as determined by qRT‐PCR. A, Let‐7b treatment results in significant decrease in the expression levels of ER‐α, ER‐ß, Cyp19a, KRAS 4A, KRAS 4B and IL‐6, relative to the control group. B, Expression of IGF‐1, cyclin‐D1, MMP‐2, TLR‐4 and IL‐10 were unchanged, and no statistical significance was found. Data are presented as mean percentage ± SEM.; *P < 0.05

The effect of Let‐7b treatment on expression of genes that are involved in endometriosis was determined by qRT‐PCR in the lesions and compared with expression in the control group. We observed decreased expression of several genes known to mediate endometriosis growth or endometriosis‐associated inflammation. Expression of ER‐α, ER‐β, Cyp19a, KRAS 4A, KRAS 4B and IL‐6 was all decreased in the Let‐7b treatment group compared to control group. The quantitative decrease in gene expression is 11.7‐fold (*P* = 0.02) for ER‐α, 3.3‐fold (*P* = 0.02) for ER‐β, 8.9‐fold (*P* = 0.02) for Cyp19a, 22.6‐fold (*P* = 0.02) for IL‐6, 10.9‐fold (*P* = 0.02) for KRAS 4A and 4.6‐fold (*P* = 0.04) for KRAS 4B in Let‐7b‐treated group compared to untreated group as shown in Figure 2A. Expression levels of the IGF‐1, cyclin‐D1, MMP‐2, TLR‐4 and IL‐10 were unchanged between the two groups (*P* > 0.05) as shown in Figure 2B.

**Figure 3:**
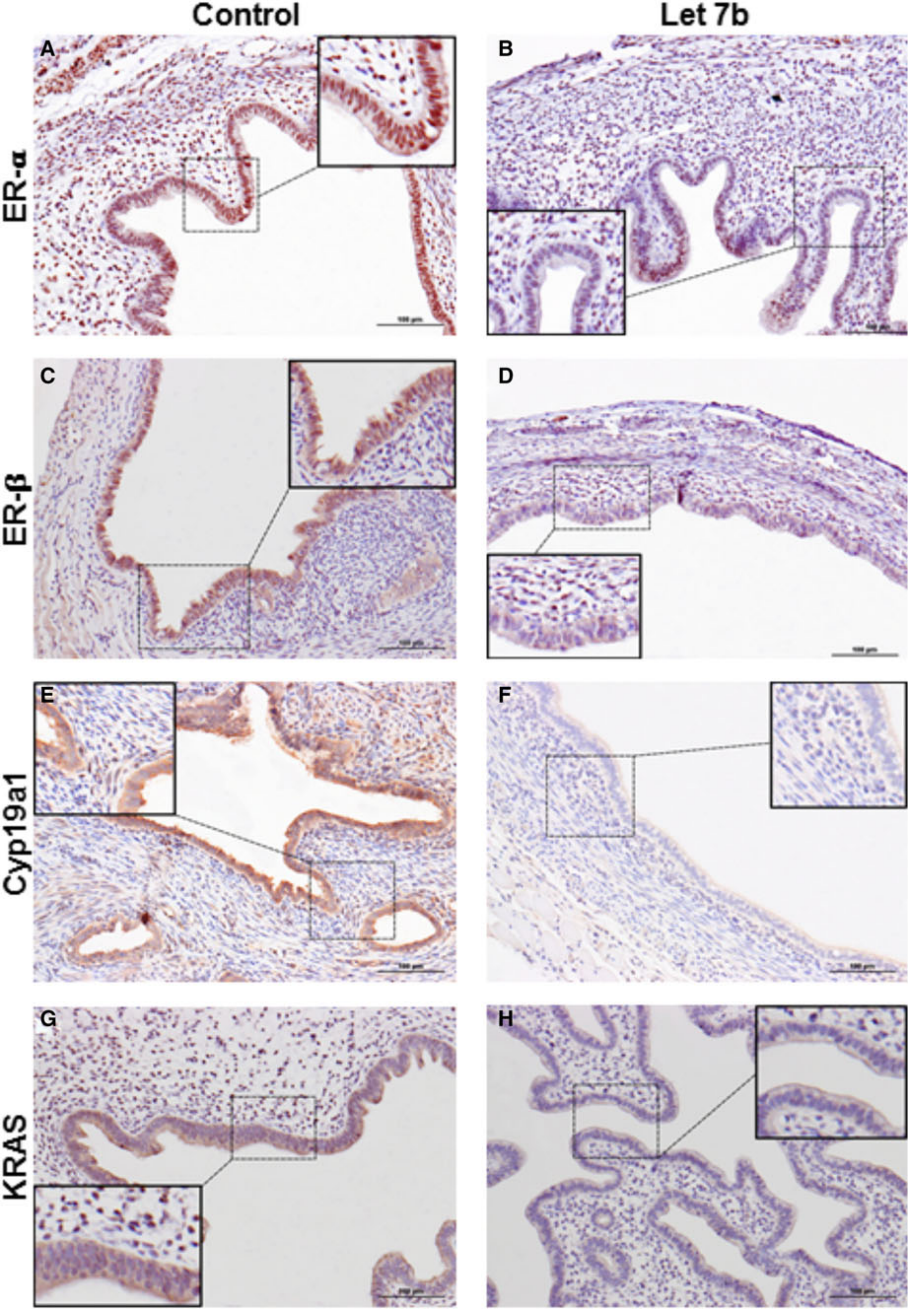
Representative images of protein levels of ER‐α, ER‐β and KRAS by immunohistochemical staining. ER‐α protein (A,B), ER‐β protein (C,D), and Cyp19a protein (E,F) and KRAS protein (G,H). Protein levels were significantly decreased in microRNA Let‐7b treated group compared to the control group. Additional panels in each image show the higher magnification of the immunohistochemical staining that shows the nuclear and/or cytoplasmic localization of the respective protein. (magnification, ×20, Scale bar = 100 μm)

The differential protein expression level of the genes that were significantly altered in the Let‐7b treatment group was confirmed by immunohistochemical analysis. Decreased epithelial and stromal cell nuclear staining and intensity of ER‐α staining were observed in the Let‐7b treatment group (Figure 3A & B). Similarly, the intensity of ER‐ß nuclear staining in epithelial cells was significantly decreased in Let‐7b treatment group compared to the untreated group (Figure 3C & D). Additionally, reduced cytoplasmic expression of Cyp19a1 (Figure 3E & F) and KRAS (Figure 3G & H) in epithelial cells was determined in Let‐7b treatment group compared to the untreated group.

## 4 DISCUSSION

We identify a novel, nonhormonal therapy for endometriosis that is based on differential expression of a miRNA in endometriosis. The treatment of endometriosis by intraperitoneal injections of miRNA Let‐7b in a murine model appears very promising given that the altered expression of this miRNA is also a key driver of human endometriosis.^21^ Delivery of miRNA Let‐7b intraperitoneally in mice showed decreased lesion growth and decreased levels of genes that have a role in the pathophysiology of endometriosis. These results support our hypothesis that miRNAs, especially Let‐7b, may be useful as a therapeutic agent/s for the treatment of endometriosis.

Macroscopic evaluation of lesions is one of the tests for determining effectiveness of endometriosis treatment. Therefore, lesions were initially compared using total volume. However, most of the lesions were compromised of fluid‐filled cystic structures in this rodent endometriosis model. Histologic tissue evaluation proved a more accurate technique than measuring lesion size. Therefore, we speculate that the fluid containing portions of the cystic lesions would also resolve over time as the endometriosis‐mediated fluid production and inflammation dissipate.

Let‐7b has tumour suppressor activity and regulates cell cycle.^10^ Suppression of Let‐7 family members has been reported in many cancers.^21^, ^22^, ^23^ KRAS, a potent proto‐oncogene,^24^, ^25^ is mutated in a wide variety of human malignancies^26^, ^27^, ^28^, ^29^, ^30^, ^31^ and up‐regulated in endometriosis. Activation of mutated KRAS in transplanted endometrium triggers endometriosis in mice.^32^ Similarly, we have shown that a polymorphism of a Let‐7‐binding site in KRAS 3′‐UTR causes abnormal KRAS expression as well as increased proliferation and invasion in endometriosis.^16^ Let‐7b regulates KRAS expression by binding to one or more of 10 Let‐7 complementary sites (LCS) in the 3′‐UTR of the KRAS gene.^33^ Here, we show that both the KRAS 4A and 4B isoforms as well as total KRAS protein expression levels were diminished in the Let‐7b treatment group.

Endometriosis is an oestrogen‐dependent disease, and oestrogen receptors ER‐α and β have a role in endometriosis.^34^ Breast cancer is another oestrogen‐dependent disease where Let‐7 also affects ER expression.^35^, ^36^ Decreased Let‐7 family miRNAs were demonstrated in ER‐α‐positive breast cancers, and there was inverse correlation between several Let‐7 family members and ER‐α expression levels.^37^ Additionally, Let‐7 a, b and i mimics transfected into MCF7 breast cancer cells reduced ER‐α transcription activity; the most effective was Let‐7b. ER‐α is a Let‐7 target where Let‐7 represses oestrogen signalling.^37^ Further, down‐regulation of Let‐7 miRNAs has been reported in breast tumour‐initiating cells (BT‐IC), and restoration of Let‐7 in these cells caused reduced proliferation and mammosphere formation in vitro as well as tumour formation and metastasis in NOD/SCID mice.^38^ We, previously identified stem cells in endometrium and endometriosis.^39^, ^40^, ^41^ It will be interesting to see whether Let‐7 also regulates stem cells in endometriosis in a similar manner to regulation of BT‐IC in breast cancers. In our model, ER‐α expression was more strongly suppressed than ER‐β with Let‐7b miRNA suggesting that Let‐7 blocks oestrogen stimulation in the treatment of endometriosis. These data suggest a targeted means of blocking sex steroid action in endometriosis without the systemic side effects of whole‐body oestrogen deprivation.

Aromatase P450 has an essential role in oestrogen synthesis and has been demonstrated to regulate local oestrogen production in endometriosis.^42^ Shibahara et al^43^ showed that high Let‐7f expression was significantly correlated with low aromatase protein levels in primary breast cancer stromal cells. They also identified a Let‐7f binding site in Cyp19a1, indicating that the aromatase gene is a direct target of Let‐7f.^43^ We demonstrated similar results in endometrial stromal cells from endometriosis patients and in Ishikawa cells.^17^ In our study, significantly increased Let‐7b and Let‐7f expression levels were determined after aromatase inhibitor treatment. Further, decreased aromatase expression and reduced endometrial cell migration were shown after Let‐7f mimic transfection.^17^ High levels of Let‐7b after aromatase inhibitor treatment indicated that Let‐7b has a role in aromatase regulation and decreased aromatase levels after Let‐7b treatment supported that Let‐7b treatment reduces local oestrogen production and action.

Endometriosis is also a chronic inflammatory disease, and macrophages have a principal role in this inflammatory process. An increase in M1 type macrophages is seen in endometriosis.^44^ Let‐7b may regulate inflammation through its known target gene TLR‐4, which regulates M1 macrophage response.^45^ We observed a trend towards decreased levels of TLR‐4 in the Let‐7b treatment group compared to controls. Another inflammatory marker, IL‐6, levels were significantly suppressed in the Let‐7b treatment group. These results suggest that Let‐7b treatment does reduce inflammation associated with endometriosis.

In this study, a local treatment route was used for miRNA Let‐7b treatment. The effective delivery of the oligonucleotides to target cells after systemic administration is not very effective as they are eliminated from the bloodstream by hepatic degradation. Therefore, many of the systemic oligonucleotide treatment studies have focused on liver disease.^46^ Various types of oligonucleotide carriers have been used to resolve these problems; however, none of them has fully accomplished targeted oligonucleotide delivery.^47^ The most effective oligonucleotide‐based drugs approved by the U.S. Food and Drug Administration (FDA) are designed for intravitreal injection where local delivery is very effective.^48^ We suggest that this drug would work best as an intraperitoneal therapy delivered at the time of surgery or by injection. Further, dose response and safety studies will be required prior to human application.

In conclusion, microRNA Let‐7b treatment of endometriosis resulted in decreased oestrogen signalling (ER and Cyp19A1), decreased KRAS and decreased inflammatory signalling (IL‐6). The pleiotropic effects of Let‐7b treatment suggest that multiple complementary mechanisms were responsible for the actions of Let‐7b in endometriosis. These numerous effects suggest the potential for a more comprehensive endometriosis therapy without the systemic effects, a common feature of current drugs.

## CONFLICT OF INTEREST

The author(s) declared no potential conflict of interests with respect to the research, authorship and/or publication of this article.
